# Nucleosome Clustering as a Biomarker and Mechanistic Switch for Reprogramming Cells

**DOI:** 10.3390/cells15020113

**Published:** 2026-01-08

**Authors:** Zhaoyuan Xu, Yinzhi Xu, Lidan You, Hiroki Yokota

**Affiliations:** 1 Weldon School of Biomedical Engineering, Purdue University Indianapolis, Indianapolis, IN 46202, USA; 2 Department of Mechanical and Materials Engineering, Queen’s University, Kingston, ON K7L 3N6, Canada; you.lidan@queensu.ca; 3 Indiana University Melvin and Bren Simon Comprehensive Cancer Center, Indianapolis, IN 46202, USA; 4 Department of Anatomy, Cell Biology and Physiology, Indiana University School of Medicine, Indianapolis, IN 46202, USA; 5 Indiana Center for Musculoskeletal Health, Indiana University School of Medicine, Indianapolis, IN 46202, USA

**Keywords:** vibration, electrical fields, optical pulses, nucleosome clustering, Piezo1, OPN4, iTS cells

## Abstract

Chromatin architecture is highly dynamic, undergoing nanoscale rearrangements throughout the cell cycle and in response to environmental cues. In this study, we employed high-resolution stochastic optical reconstruction microscopy (STORM) to visualize chromatin organization and cellular plasticity at the nanoscale in two osteosarcoma cell lines, U2OS and MG63. To promote a tumor-suppressive bone microenvironment, we applied three biophysical modalities, namely mechanical vibration, electrical stimulation, and optical pulses, each previously linked to altered tumor behavior by reprogramming cells and generating induced tumor-suppressing (iTS) cells. These stimuli enlarged nuclear size and disrupted nuclear envelope integrity, as revealed by increased surface roughness. Critically, all three modalities transiently scattered nucleosome clusters, indicating chromatin decondensation as a hallmark of iTS cell generation. iTS cells exhibited elevated expression of histone demethylases lysine demethylase 3A (KDM3A) and lysine demethylase 4 (KDM4), accompanied by reduced levels of trimethylated histone H3 lysine 9 (H3K9me3). Consistently, pharmacological agents—Trichostatin A as a histone deacetylase inhibitor and chaetocin as a histone methyltransferase inhibitor—induced nucleosome scattering and converted U2OS cells into iTS cells, whose conditioned media exerted tumor-suppressive effects. Our findings highlight nucleosome clustering as a key epigenetic feature responsive to both biophysical and chemical cues, underscoring its role in microscale chromatin remodeling and reprogramming of the tumor microenvironment.

## 1. Introduction

Chromatin architecture functions not merely as a passive structural scaffold for organizing the genome [1,2], but as an active and dynamic regulator of cellular identity, gene expression, and functional plasticity [3]. In the context of cancer biology, aberrations in nucleosome positioning, histone modifications, and chromatin condensation are increasingly recognized as defining features of malignant transformation [4]. These epigenetic alterations contribute to the reprogramming of transcriptional networks and metabolic pathways, enabling tumor cells to evade normal regulatory constraints, adapt to hostile microenvironments, and sustain uncontrolled proliferation [5].

Building on this paradigm, we investigated whether chromatin remodeling triggered by biophysical stimulation is mechanistically linked to the reprogramming of osteosarcoma cells into tumor-suppressive phenotypes. Specifically, we asked whether the tumor-suppressive properties of induced tumor-suppressing (iTS) cells [6,7] are causally associated with alterations in chromatin architecture that activate cellular metabolic pathways. We hypothesized that nucleosome scattering, a hallmark of relaxed chromatin structure, facilitates the generation of iTS cells, and that their conditioned medium (CM) functions as a distinct tumor-suppressive agent. To test this, we employed stochastic optical reconstruction microscopy (STORM) [8] to visualize changes in nucleosome clustering and nuclear envelope morphology in response to low-intensity vibration (LIV) [9], electrical stimulation (ES) [10], and optical pulses (OPs), three biophysical modalities previously shown to activate cell metabolism. We also examined transient chromatin decondensation and disrupted nuclear architecture, both indicative of heightened metabolic activity and cellular plasticity.

To further probe the mechanistic relevance of chromatin relaxation in the induction of tumor-suppressive phenotypes, we employed trichostatin A (TSA), a well-characterized histone deacetylase (HDAC) inhibitor [11], known to promote chromatin loosening through increased histone acetylation. We also employed chaetocin, a methyltransferase inhibitor, which similarly led to nucleosome scattering [12]. Taxol was employed as a negative control, which is a chemotherapeutic agent for killing cancer cells [13]. We addressed whether TSA treatment recapitulated the effects of biophysical stimulation, both in terms of nucleosome scattering and the generation of iTS cells, as evidenced by similar morphological and functional outcomes. We focused on the regulation of H3K9me3 (histone 3 lysine 9 trimethylated isoform) [14] by KDM3A and KDM4, histone lysine demethylases specific to H3K9 [15]. This pharmacological mimicry supports the notion that nucleosome scattering represents a convergent epigenetic mechanism underlying the transition to tumor-suppressive states. Notably, however, excessive scattering by histone modifications turned out to be cytotoxic.

In this study, we employed two osteosarcoma cell lines, U2OS [16] and MG63 [17], to investigate whether chromatin remodeling induced by biophysical and chemical perturbations is mechanistically linked to the reprogramming of cancer cells into induced tumor-suppressing (iTS) phenotypes. While previous studies on iTS cell generation and mechanotransduction have largely focused on signaling pathways, metabolic activation, and secreted tumor-suppressive factors, the contribution of higher-order chromatin organization has remained insufficiently explored. Here, we move beyond treating nucleosome clustering as a descriptive epigenetic marker and instead examine whether controlled nucleosome scattering represents a permissive mechanistic switch that facilitates iTS cell generation in response to diverse biophysical cues. Osteosarcoma provides a particularly informative model for this investigation, as osteosarcoma cells exhibit pronounced nuclear atypia, epigenetic plasticity, and sensitivity to mechanical and metabolic perturbations, reflecting their origin within a mechanically dynamic bone microenvironment. Collectively, these considerations position nucleosome topology as both a sensitive biomarker and a functional regulator of tumor-suppressive reprogramming.

## 2. Materials and Methods

### 2.1. Cell Culture and MTT Assay

U2OS and MG63 cell lines were cultured in DMEM media supplemented with 10% fetal bovine serum and antibiotics (50 U/mL penicillin and 50 μg/mL streptomycin). Cells were maintained at 37 °C in a humidified incubator with 5% CO_2_. Cell viability was assessed using the MTT assay, which measures metabolic activity as an indicator of cell health. After incubation with MTT reagent, the resulting formazan product was quantified by measuring absorbance at 570 nm using a multi-well spectrophotometer.

### 2.2. Application of Biophysical Stimuli

To investigate cellular responses to mechanical and electromagnetic cues, U2OS and MG63 osteosarcoma cells were subjected to three distinct biophysical stimulations. Low-intensity vibration (LIV) was administered using a custom-engineered platform following established protocols [9], with cells exposed to vertical vibrations at a frequency of 90 Hz and a peak acceleration of 0.7 g for 20 min per session. A total of two vibration sessions were applied, with an interval of 3 h between sessions. For electrical stimulation, a pair of platinum electrodes (240 µm diameter) was placed 2 cm apart within the culture medium of standard dishes, replicating previously validated conditions [10]. Electrical stimulation was applied at 0.1 V with a pulse frequency of 100 Hz for 1 h under standard incubation conditions (37 °C, 5% CO_2_). Optical stimulation employed high-intensity LEDs (LED4D022, Thorlabs, Newton, NJ, USA) emitting monochromatic light at 470 nm (blue), 530 nm (green), and 625 nm (red). Light pulses were modulated using a dedicated LED driver (DC4104, Thorlabs) in conjunction with a programmable function generator (AFG-2005, GW Instek, New Taipei City, Taiwan). Cells were exposed to pulsed illumination at a frequency of 10 Hz for 1 h, with illuminance set at 5000 lux for blue and green light and 100 lux for red light. Illuminance was measured in lux using a calibrated digital lux meter (LX1330B, Thoursandshores Inc., Newark, CA, USA).

### 2.3. Western Blot Analysis

Cells were lysed in RIPA buffer supplemented with protease (Santa Cruz Biotechnology, Dallas, TX, USA) and phosphatase inhibitors (Calbiochem, San Diego, CA, USA). Protein concentrations were measured using a BCA assay (Thermo Fisher Scientific, Waltham, MA, USA). Equal amounts of protein were separated on 10–15% SDS-PAGE gels and transferred to PVDF membranes (Millipore, Burlington, MA, USA). Membranes were blocked for 1 h (Bio-Rad, Hercules, CA, USA), incubated overnight with primary antibodies, and then with HRP-conjugated secondary antibodies for 45 min (Cell Signaling, Danvers, MA, USA). Signals were detected using SuperSignal West Femto substrate (Thermo Fisher Scientific) and quantified with a luminescent image analyzer (LAS-3000, Fujifilm, Tokyo, Japan). Antibodies against Lamin A/C, OPN4 (Santa Cruz Biotechnology), Piezo1, KDM3A (Proteintech, Rosemont, IL, USA), H3K9me3 (Active Motif, Carlsbad, CA, USA), KDM4 (Cell Signaling), and β-actin (Sigma-Aldrich, St. Louise, MO, USA) were used. The grayscale intensity of Western blot bands was quantified using ImageJ software (Version 1.53t, National Institutes of Health, Bethesda, MD, USA). The intensity values were normalized to β-actin.

### 2.4. RNA Interference

RNA interference was carried out in U2OS cells to silence the expression of Piezo1 and OPN4. Cells were transfected with the selected siRNAs using Lipofectamine^®^ 3000 (L300015; Thermo Fisher Scientific, Waltham, MA, USA), along with a negative control siRNA (4390843; Thermo Fisher Scientific, Waltham, MA, USA), following the manufacturer’s protocol.

### 2.5. Live/Dead Cell Staining Assay

U2OS cells were seeded and treated with 5 μM Taxol in DMEM for 48 h to induce cytotoxic stress, while untreated cells served as controls. Live and dead cells were subsequently visualized using the LIVE/DEAD™ Cell Imaging Kit (R37601; Invitrogen, Carlsbad, CA, USA), which employed Calcein-AM (488 nm) to label viable cells and BOBO-3 iodide (570 nm) to label membrane-compromised dead cells. Staining was performed according to the manufacturer’s instructions. Briefly, cells were incubated with the staining reagents for 20 min at 37 °C in the dark, followed by immediate imaging under a fluorescence microscope. Live cells were identified by green fluorescence, whereas dead cells were identified by red fluorescence. For each condition, multiple random fields were imaged.

### 2.6. Stochastic Optical Reconstruction Microscopy (STORM)

To evaluate changes in nucleosome organization following biophysical stimulation and chemical treatments, we performed STORM imaging using U2OS cells expressing Halo-tagged histone H2B. Cells were incubated with the JF646 HaloTag ligand (GA1120; Promega, Madison, WI, USA) in STORM buffer (Supplementary Table S1) [18]. JF646 selectively binds to Halo-tagged H2B and is excitable with a 640 nm laser. Imaging was conducted using an Olympus IX83 microscope (IX3-LBD; Olympus, Hachioji, Japan) equipped with a 60 × 1.25 NA oil-immersion objective lens and illuminated with a 20 mW 640 nm laser (Excelitas Technologies, Wiesbaden, Germany). Image acquisition was performed using an ORCA-Fusion sCMOS camera (C14440-20UP; Hamamatsu Photonics, Hamamatsu, Japan), controlled via Micro-Manager 2.0.

Approximately 2000 sequential fluorescence images were acquired (100 frames per second, 2000 frames). Each fluorescent signal appeared as a point spread function (PSF), representing how light from a single molecule is distributed in the image. By analyzing these PSFs, the high-resolution location of each fluorescent molecule was predicted, enabling the reconstruction of chromatin architecture. Nucleosome clustering was quantified using the “L(r)–r” function [19], a spatial statistic derived from Besag’s L-function. This metric measures how many nucleosomes are located within a given distance (r) of each other. Higher values of “L(r)–r” indicate increased nucleosome clustering, suggesting a more compact chromatin state.

### 2.7. Immunofluorescence (IF) Staining

U2OS cells were cultured in 3.5 cm glass-bottom dishes and subjected to designated treatments, followed by three PBS washes (5 min each). Cells were fixed with 4% paraformaldehyde for 10 min, washed again (3 × 5 min), permeabilized with 0.1% Triton X-100 in PBS for 10 min, and washed with PBS (3 × 5 min). Blocking was performed with 5% BSA in PBS for 1 h, followed by triple PBS washes. Primary antibodies diluted in blocking buffer were applied overnight at 4 °C. The next day, cells were washed with PBS containing 0.1% Tween 20 (3 × 5 min), incubated with fluorophore-conjugated secondary antibodies for 1 h, and washed again (3 × 5 min in PBS + 0.1% Tween 20). Finally, cells were mounted with fluoromount-G containing DAPI and imaged immediately or stored at −20 °C.

### 2.8. PCA (Principal Component Analysis)

PCA was performed to identify correlations among nuclear size, surface roughness of the nuclear envelope, and nucleosome clustering in control and biophysically or chemically treated groups. PCA was conducted at the single-cell level, with each data point representing an individual cell. Three quantitative features—nuclear size, nuclear envelope surface roughness, and nucleosome clustering—were extracted from image analyses and used as input variables. Prior to PCA, all features were standardized by z-score normalization to ensure equal weighting. PCA was performed using standard statistical software, and analysis focused on the first and second principal component axes (PC1 and PC2) [20], which captured the majority of variance in the dataset. PCA results were visualized in both sample space (cell distribution) and feature space (feature loadings).

### 2.9. Quantification of Nuclear Envelope Surface Roughness

To assess nuclear envelope surface roughness, we delineated the outer and inner boundaries by fitting the minimum enclosing ellipse and the maximum inscribed ellipse, respectively [21]. Surface irregularity was quantified as the area difference between the two ellipses, normalized by their mean area, yielding a dimensionless ratio indicative of envelope roughness.

### 2.10. Statistical Analysis

For cell-based assays, data were obtained from three or four biologically independent experiments, each performed under consistent experimental conditions. Quantitative results are presented as the mean ± standard deviation (SD), unless otherwise specified. Statistical comparisons between two groups were performed using an unpaired, two-tailed Student’s *t*-test. A *p*-value of less than 0.05 was considered statistically significant. In all figures, statistical significance was denoted as follows: *p* < 0.05 with a single asterisk (*), and *p* < 0.01 with a double asterisk (**). All statistical analyses were conducted using an Excel statistical package, and assumptions of normality and equal variance were verified where applicable. Comparisons were based on predefined experimental hypotheses rather than exploratory testing across all conditions, and formal corrections for multiple testing were therefore not systematically applied. Biological replicates were defined as independent experiments performed on different days, whereas multiple cells and fields of view analyzed within each experiment were considered technical replicates.

## 3. Results

Before evaluating the effects of biophysical stimulation on U2OS cells, we conducted two control experiments using TSA (0.4 μM for 1 h) and Taxol (5 μM for 48 h) to assess their impact on nucleosome clustering. TSA induced chromatin relaxation, as evidenced by nuclear enlargement, increased nuclear envelope roughness, and reduced nucleosome clustering (Supplementary Figure S1). In contrast, Taxol triggered cell death, accompanied by roughened nuclear envelopes and enhanced nucleosome clustering (Supplementary Figure S2). These findings demonstrate that TSA and Taxol exert opposing effects on chromatin architecture, underscoring the critical role of pharmacologically induced chromatin remodeling.

### 3.1. Mechanical Vibration (LIV) Induced Chromatin Loosening and Nuclear Envelope Remodeling

**Figure 1:**
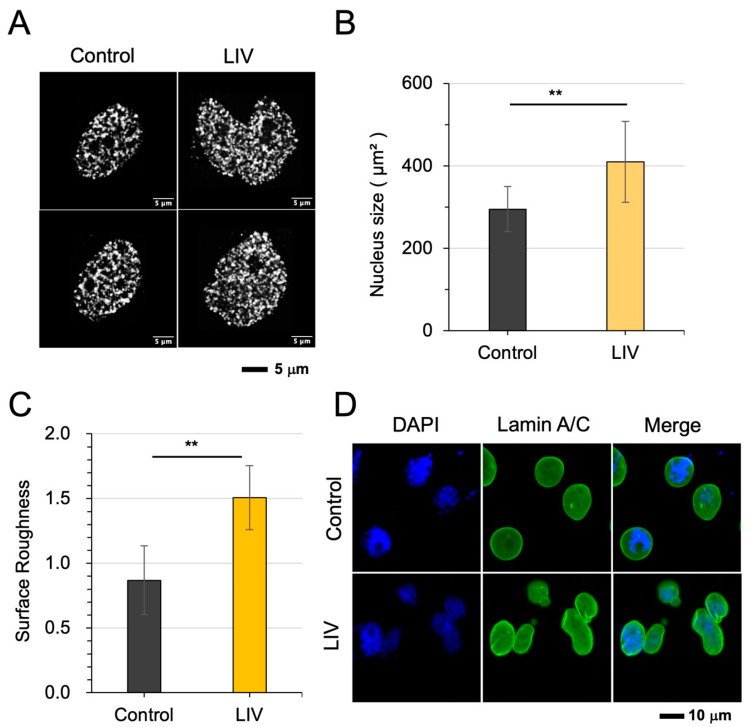
Mechanical vibration induces chromatin loosening and nuclear envelope remodeling in U2OS cells. (**A**,**B**) Representative STORM images showing nuclear morphology before and after mechanical vibration, showing enlarged nucleus size. (**C**) Quantification of nuclear envelope roughness, demonstrating an increase following mechanical vibration. (**D**) Lamin A/C-stained fluorescent images, showing the deformation of the nuclear envelope by mechanical vibration. The double asterisks (**) indicate *p* < 0.01.

We first examined whether low-intensity mechanical vibration alters nuclear architecture and chromatin organization. LIV was applied as vertical oscillations at 90 Hz with an acceleration level equivalent to 0.7× Earth’s gravity. STORM imaging revealed nanoscale alterations in chromatin organization (Figure 1A). Specifically, nuclei exhibited a modest increase in size (Figure 1B) and an elevation in nuclear envelope roughness (Figure 1C). Representative Lamin A/C-stained fluorescent images before and after LIV exposure revealed the deformation in the nuclear morphology (Figure 1D). In particular, quantitative analysis of nucleosome clustering demonstrated a significant decrease in chromatin compaction following LIV stimulation (Supplementary Figure S3A). The MG63 cells also showed consistent responses with the U2OS cell line (Supplementary Figure S4). Moreover, principal component analysis (PCA) suggested an interrelationship among nucleosome clustering and nuclear size and envelope surface roughness (Supplementary Figure S3C), suggesting a coordinated remodeling of the nuclear structure in response to mechanical cues.

### 3.2. ES Induced Chromatin Dispersion and Nuclear Remodeling

**Figure 2:**
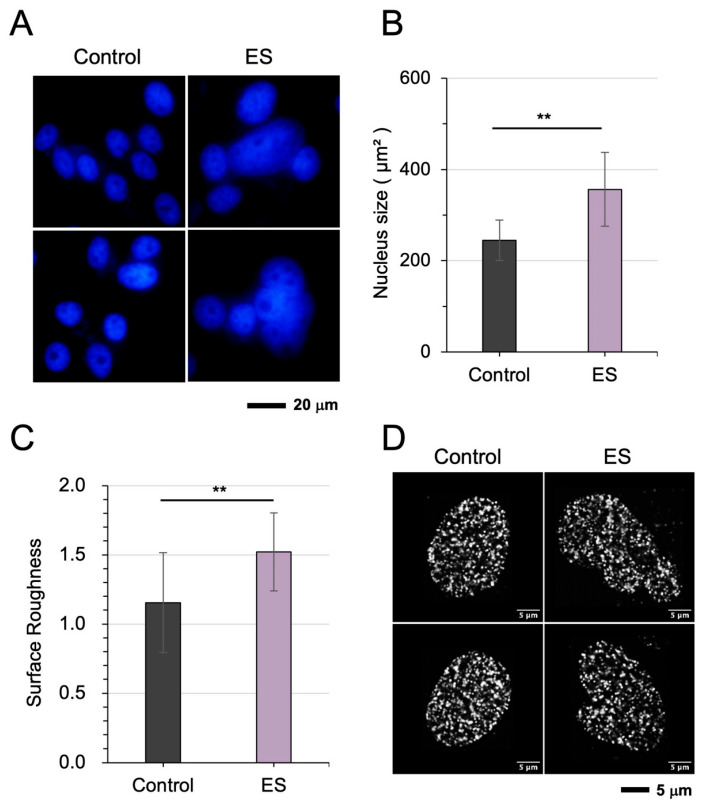
Electrical stimulation (ES) promotes chromatin dispersion and nuclear remodeling in U2OS cells. (**A**) Representative DAPI-stained images of nuclei before and after ES, illustrating chromatin dispersion. (**B**,**C**) Quantitative analysis, showing an increase in nuclear size and nuclear envelope surface roughness following ES. (**D**) High-resolution STORM images of nuclei before and after ES, revealing changes in chromatin organization at the nanoscale. The double asterisks (**) indicate *p* < 0.01.

We next examined whether electrical stimulation induces nuclear and chromatin remodeling comparable to that observed under mechanical vibration. ES (0.1 V/cm at 100 Hz for 1 h) was applied to U2OS cells. Similar to the effects observed with LIV, representative DAPI-stained images demonstrated pronounced chromatin dispersion following ES (Figure 2A). Quantitative analysis further revealed a significant increase in nuclear size and a concomitant increase in nuclear envelope surface roughness post-stimulation (Figure 2B,C). High-resolution STORM images provided additional insight into nanoscale chromatin architecture, showing a reduction in the “L(r)–r” value after ES, indicative of diminished nucleosome clustering (Figure 2D and Supplementary Figure S3B). While PCA of nuclear size, envelope roughness, and nucleosome clustering suggests that ES-induced alterations in these features were not identical to the patterns in the LIV responses (Supplementary Figure S3D), the clustering feature was similarly positioned on the corner of the PC1 and PC2 axes. ES induced the observed chromatin and nuclear remodeling also in the MG63 cell line (Supplementary Figure S5).

### 3.3. Optical Pulses Modulated Chromatin Clustering in a Wavelength-Dependent Manner

**Figure 3:**
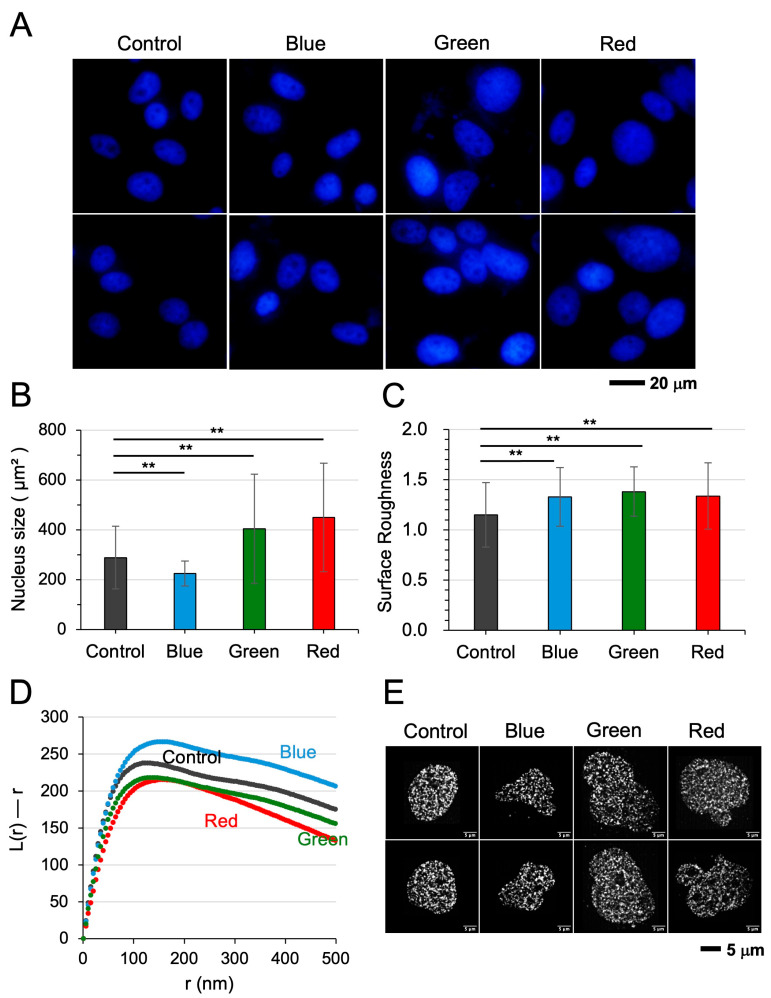
Chromatin clustering is modulated by optical pulses in a wavelength-dependent manner. (**A**,**B**) DAPI-stained images of nuclei and quantification of nuclear size changes following exposure to red, green, or blue light. Red and green light increased nuclear size, whereas blue light decreased it. (**C**) All three wavelengths increased the surface roughness of the nuclear envelope. (**D**) Red and green light pulses reduced nucleosome clustering, while blue light enhanced clustering. (**E**) High-resolution STORM images of nuclei before and after irradiation with red, green, or blue light, illustrating wavelength-specific effects on chromatin organization. The double asterisks (**) indicate *p* < 0.01.

To determine whether optical stimulation induces chromatin remodeling in a wavelength-dependent manner, we examined the effects of red, green, and blue light pulses on nuclear architecture. DAPI-stained images and their quantitative analysis revealed that nuclear size responded differentially to light wavelength: red and green light pulses induced nuclear enlargement, whereas blue light led to a reduction in nuclear size (Figure 3A,B). Despite these divergent effects on size, all three wavelengths consistently elevated nuclear envelope surface roughness (Figure 3C). Strikingly, chromatin organization was modulated in a wavelength-specific manner. Red and green light pulses reduced nucleosome clustering, while blue pulses enhanced it, as evidenced by changes in the “L(r)–r” metric (Figure 3D). In the other osteosarcoma cell line, MG63, red and green light pulses (but not blue light pulses) also led to nucleosome scattering (Supplementary Figure S6). High-resolution STORM imaging further illustrated the distinct chromatin remodeling patterns, highlighting the effects of red, green, and blue light on nuclear architecture (Figure 3E).

### 3.4. The Effects of Green Light Pulses on Nucleosome Clustering and Nuclear Morphology Were Transient

**Figure 4:**
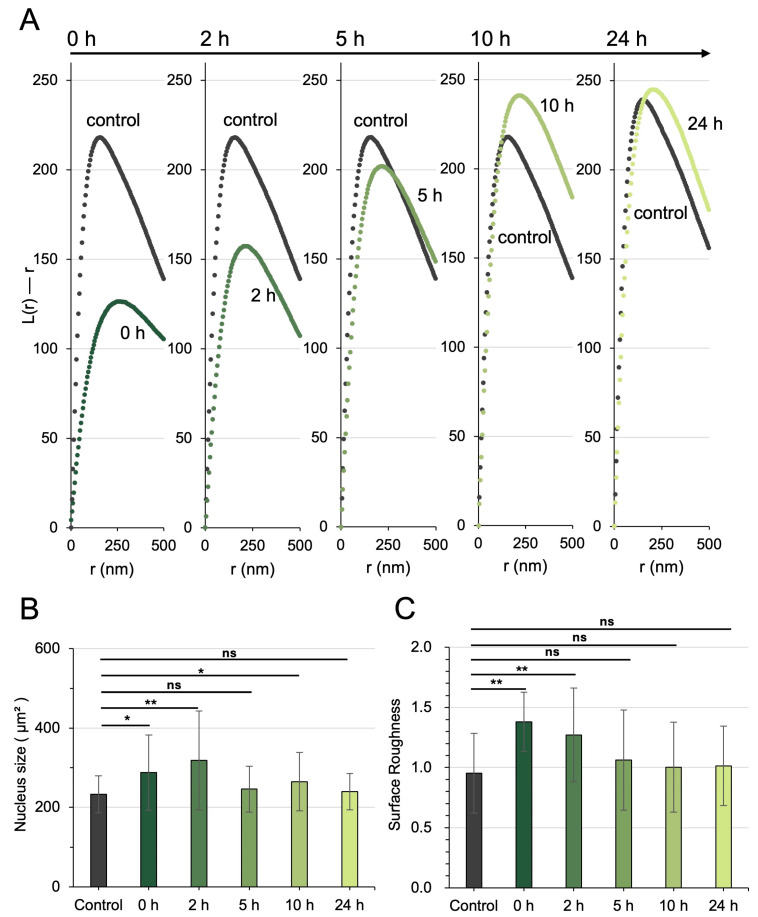
Transient effects of green light pulses on nucleosome clustering and nuclear morphology. (**A**) Time-dependent changes in the “L(r)–r” value over 24 h, showing a transient reduction in nucleosome clustering following green light exposure. (**B**,**C**) Temporal dynamics of nuclear size and nuclear envelope surface roughness in response to green light pulses. Both nuclear size and envelope roughness recovered within 24 h. The single (*) and double (**) asterisks indicate *p* < 0.05 and 0.01, respectively; “ns” denotes not significant.

To determine whether the effects of green light pulses on chromatin architecture were transient or sustained, we monitored nucleosome clustering and nuclear morphology over 24 h. Time-course analysis of the “L(r)–r” metric revealed a transient decrease in nucleosome clustering following green light exposure, with values gradually returning to baseline (Figure 4A). In parallel, we observed dynamic changes in nuclear morphology. Nuclear size initially increased but returned to pre-stimulation levels within 24 h (Figure 4B). Consistently, the surface roughness of the nuclear envelopes was significantly recovered in 5 h (Figure 4C).

### 3.5. PIEZO1 Mediated Nuclear Responses to LIV and ES

**Figure 5:**
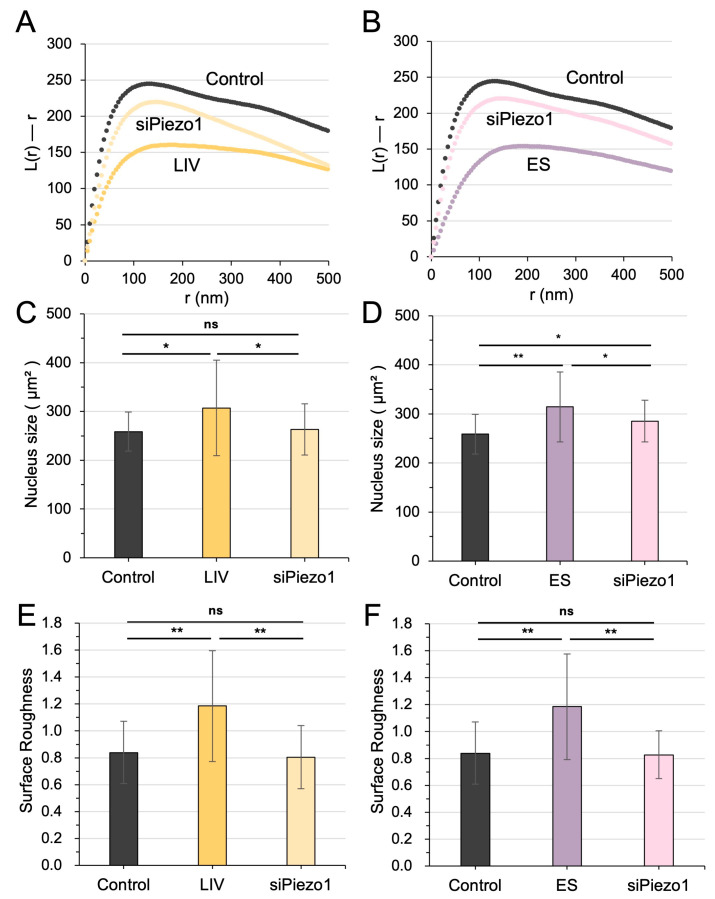
PIEZO1 mediates nuclear responses to mechanical vibration and electrical stimulation (ES). (**A**,**B**) Silencing of PIEZO1 via siRNA significantly attenuates nucleosome scattering induced by mechanical vibration and ES, respectively. (**C**,**D**) PIEZO1 knockdown also reduces the increase in nuclear size triggered by mechanical vibration and ES, respectively. (**E**,**F**) The increase in the surface roughness of the nuclear envelope, caused by mechanical vibration and ES, was rescued by PIEZO1 silencing. The single (*) and double (**) asterisks indicate *p* < 0.05 and 0.01, respectively; “ns” denotes not significant.

We next investigated whether PIEZO1, previously implicated in the generation of iTS cells in response to LIV and ES [9,10], also contributed to chromatin remodeling induced by these stimuli. Silencing PIEZO1 via siRNA significantly attenuated nucleosome dispersion triggered by both LIV and ES (Figure 5A,B; Supplementary Figure S7A). In addition, PIEZO1 knockdown markedly reduced the nuclear enlargement typically observed following LIV and ES (Figure 5C,D). Moreover, the increase in the surface roughness of the nuclear envelope, induced by LIV and ES, was mostly rescued by PIEZO1 silencing (Figure 5E,F). These findings suggest that PIEZO1 played a central role not only in iTS cell generation but also in mediating nuclear remodeling and nucleosome scattering in response to mechanical and electrical cues.

### 3.6. OPN4 Mediated Nuclear Responses to Optical Pulses

**Figure 6:**
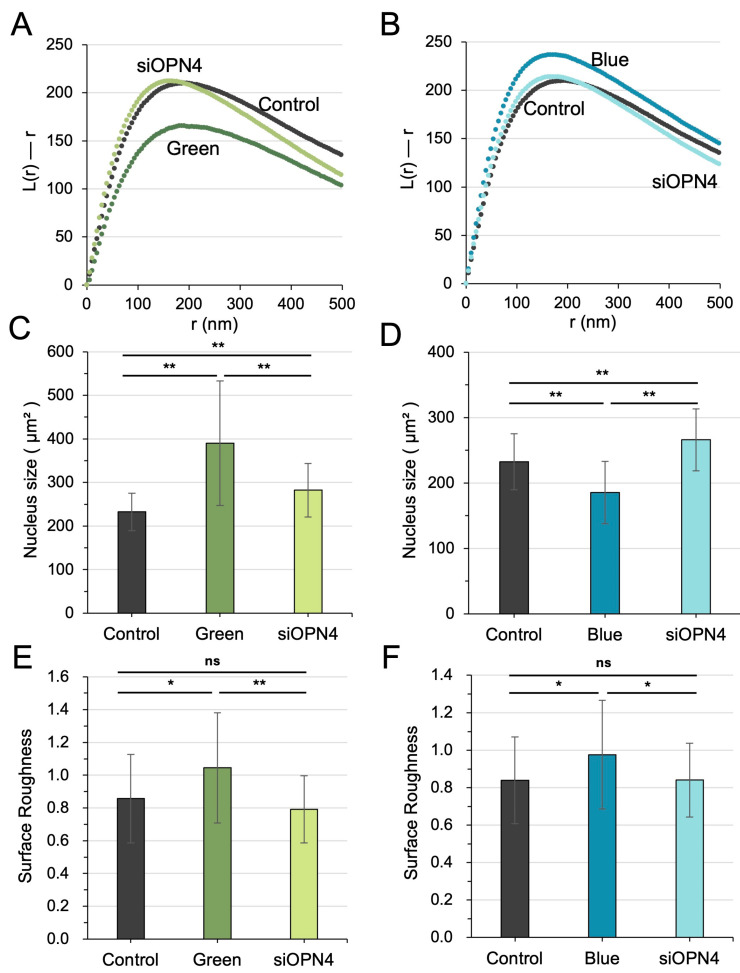
OPN4 mediates the responses of the nucleus to optical pulses. (**A**,**B**) Silencing OPN4 suppressed the alterations in the value of “L(r)–r”, which were induced by the irradiation of green and blue light pulses. (**C**,**D**) Green and blue pulses altered nuclear size, an effect that was mitigated by siOPN4. (**E**,**F**) The increase in nuclear envelope surface roughness caused by green and blue pulses was rescued by OPN4 silencing. The single (*) and double (**) asterisks indicate *p* < 0.05 and 0.01, respectively; “ns” denotes not significant.

While Piezo1 was implicated in the nuclear responses to LIV and ES, it did not contribute to the cellular responses triggered by optical pulses (Supplementary Figure S6D). Given OPN4’s established role as a photosensor, we investigated its potential involvement in mediating nucleosome scattering in response to optical stimuli. Silencing OPN4 attenuated changes in the parameter “L(r)–r” following exposure to green and blue light pulses (Figure 6A,B; Supplementary Figure S7B). In alignment with these findings, the silencing of OPN4 abrogated the light-induced temporal dynamics of nuclear size and surface roughness of the nuclear envelopes in response to green light stimulation (Figure 6C–E).

### 3.7. Tumor-Suppressive Effects of CM from Biophysically Stimulated iTS Cells

**Figure 7:**
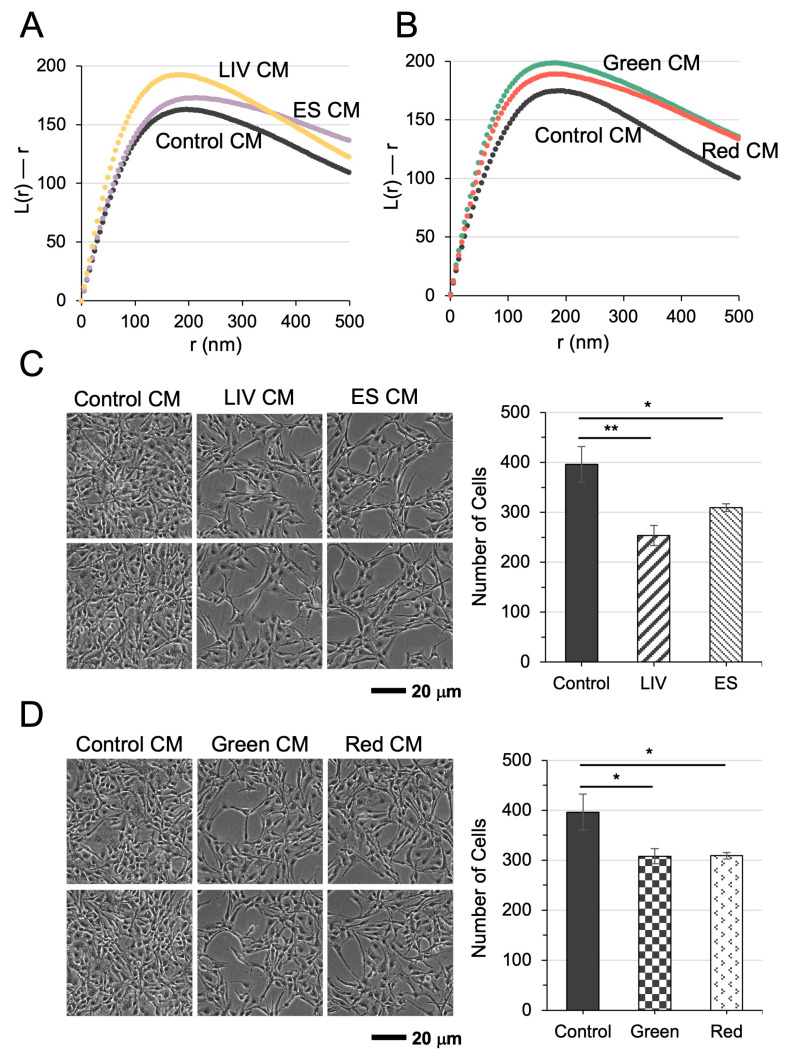
Tumor-suppressive effects of conditioned media (CM) from biophysically stimulated iTS cells. (**A**,**B**) Nucleosome clustering, assessed by the “L(r)–r” metric, increased in cells cultured with CM derived from LIV/ES or Green/Red-treated iTS cells. This enhanced clustering reflects CM-induced chromatin condensation associated with reduced cellular metabolic activity. (**C**,**D**) Optical cell images showed that CM derived from iTS cells, generated by an exposure to LIV/ES or green/red pulses, presented tumor-suppressing capabilities. The single (*) and double (**) asterisks indicate *p* < 0.05 and 0.01, respectively.

We previously demonstrated that CM derived from iTS cells exerts tumor-suppressive effects, mediated by the secretion of anti-tumor proteins [22]. In this study, we examined whether CM from iTS cells stimulated by LIV or ES could modulate chromatin architecture in a manner analogous to the chemotherapeutic agent Taxol. Quantitative analysis of nucleosome clustering, using the “L(r)–r” metric, revealed a marked increase in chromatin condensation in U2OS cells cultured with CM from LIV- or ES-treated U2OS-derived iTS cells (Figure 7A). Consistently, CM derived from iTS cells exposed to green or red optical pulses also elevated nucleosome clustering (Figure 7B), correlating with reduced metabolic activity and proliferative capacity. U2OS cells treated with these CM preparations exhibited significantly diminished proliferation (Figure 7C,D), further supporting the tumor-suppressive role of CM via chromatin remodeling. Taken together, these findings suggest that biophysical stimulation induces chromatin decondensation in iTS cells, and their secretome, reflected in the CM, acts as a potent tumor-suppressive agent by promoting chromatin condensation and metabolic suppression in recipient cancer cells.

### 3.8. Generation of iTS Cells via TSA and Chaetocin

**Figure 8:**
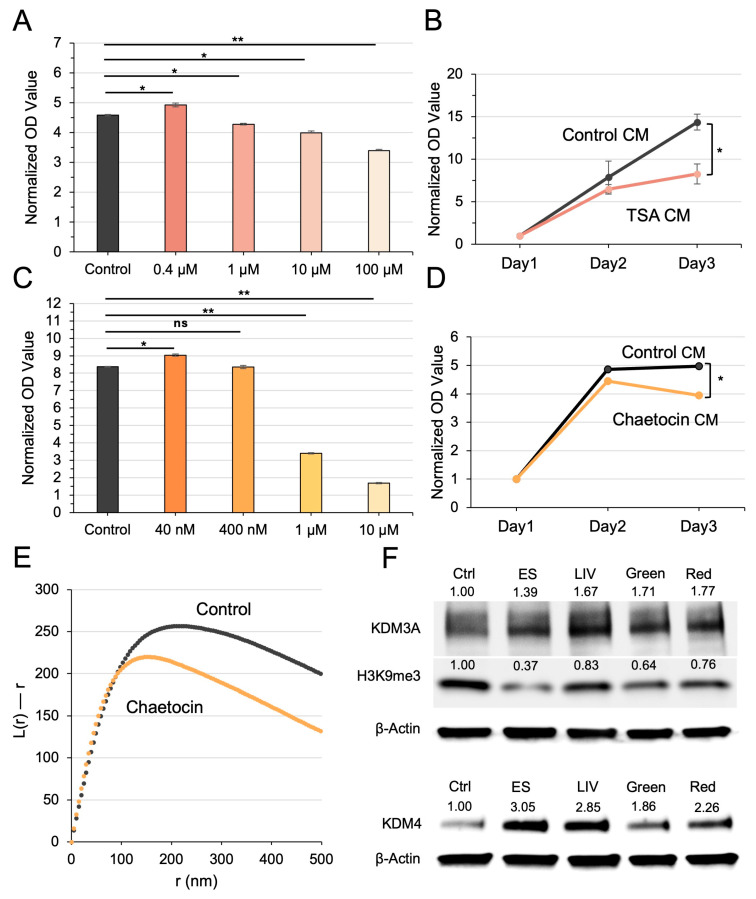
Generation of iTS cells by exposure to 0.4 μM TSA and 40 nM chaetocin. (**A**) Concentration-dependent effect of TSA on MTT-based viability of U2OS cells. (**B**) Reduction in MTT-based cellular viability of U2OS cells by TSA-treated cell-derived CM. Note, TSA concentration was 0.4 μM. (**C**,**D**) Chaetocin exhibited a similar effect on U2OS cell viability as TSA, supporting its role in modulating cell fate via epigenetic histone modification. (**E**) Chaetocin-treated U2OS cells showed an increase in nucleosome scattering. (**F**) Western blot analysis, showing an increase in KDM3A and KDM4 levels, and a decrease in H3K9me3 levels in iTS cells, which were generated in response to LIV, ES, or green/red pulses. The single (*) and double (**) asterisks indicate *p* < 0.05 and 0.01, respectively; “ns” denotes not significant.

Building on the observed link between biophysical stimuli and nucleosome scattering in iTS cell generation, we investigated whether chemical agents known to disrupt chromatin structure could similarly induce iTS cells. Specifically, we tested Trichostatin A (TSA), a histone deacetylase (HDAC) inhibitor, and Chaetocin, an inhibitor of histone methyltransferases. As anticipated, high concentrations of these agents (≥1 μM) were cytotoxic. However, lower doses, 0.4 μM TSA and 40 nM Chaetocin, enhanced MTT-based viability of U2OS cells and successfully induced iTS cell formation (Figure 8A–D). The CM derived from these chemically induced iTS cells significantly reduced the viability of naïve U2OS cells in MTT assays (Figure 8B,D), further supporting the successful reprogramming into iTS cells. Notably, the induction was dose-dependent, paralleling the effects of biophysical stimuli: excessive exposure led to cytotoxicity, whereas moderate stimulation favored iTS cell generation.

### 3.9. Elevation of KDM3A/KDM4 and Reduction in H3K9me3 in iTS Cell Reprogramming

A defining epigenetic change associated with iTS cell generation is the downregulation of H3K9me3, a repressive histone mark linked to transcriptional silencing (Figure 8F). This reduction suggests a shift toward a more permissive chromatin state, conducive to cellular reprogramming. In alignment with this observation, Western blot analysis revealed elevated levels of the histone demethylases KDM3A and KDM4, which catalyze the conversion of H3K9me3 to its less repressive forms, H3K9me2 and H3K9me1 (Figure 8F). Notably, silencing Piezo1 abolished the effect of LIV on the expression of KDM3A, KDM4, and H3K9me3, indicating that Piezo1 is required for LIV-driven generation of iTS cells. Similarly, silencing OPN4 mitigated the impact of green light pulses on these epigenetic regulators, suggesting that OPN4 mediates the photosensory pathway leading to histone methylation changes followed by the generation of iTS cells (Supplementary Figure S7C). Notably, this effect appears to be independent of PIEZO1 signaling, as PIEZO1 knockdown did not abolish the tumor-suppressive activity of conditioned media induced by green light stimulation (Supplementary Figure S7D).

**Figure 9:**
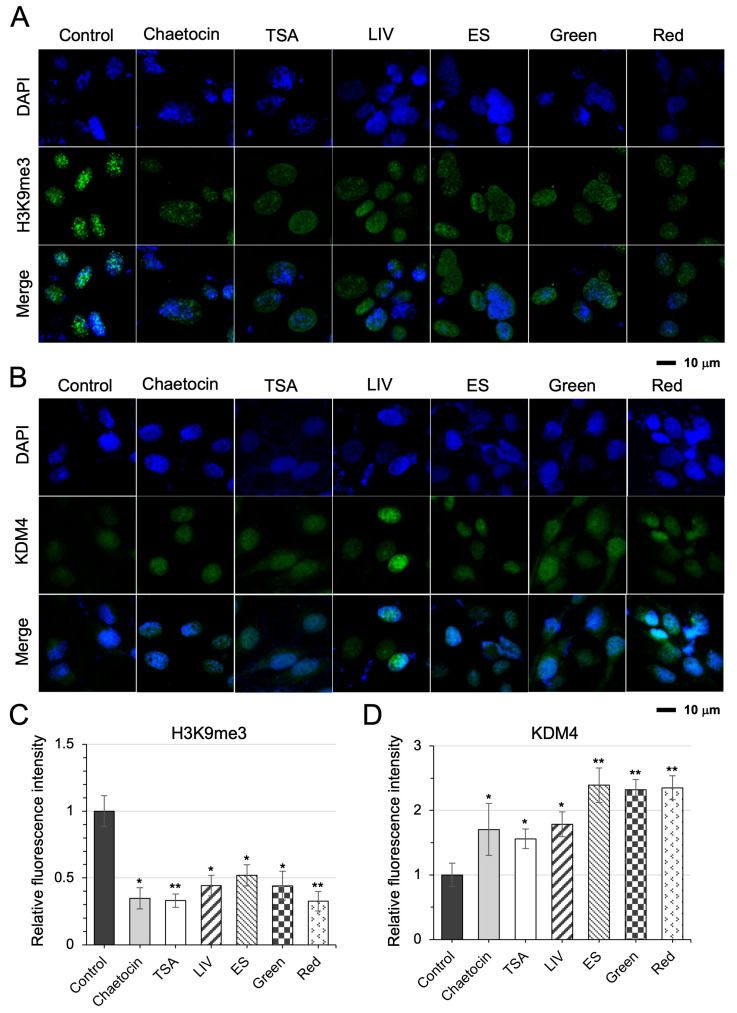
The changes in H3K9me3 and KDM4 levels by immunofluorescence staining. (**A**,**B**) Decrease in the level of H3K9me3 and an increase in the level of KDM4 in U2OS cells in response to the 6 different treatments to generate iTS cells. (**C**,**D**) Relative fluorescence intensity of H3K9me3 and KDM4. The single (*) and double (**) asterisks indicate *p* < 0.05 and 0.01, respectively.

**Figure 10:**
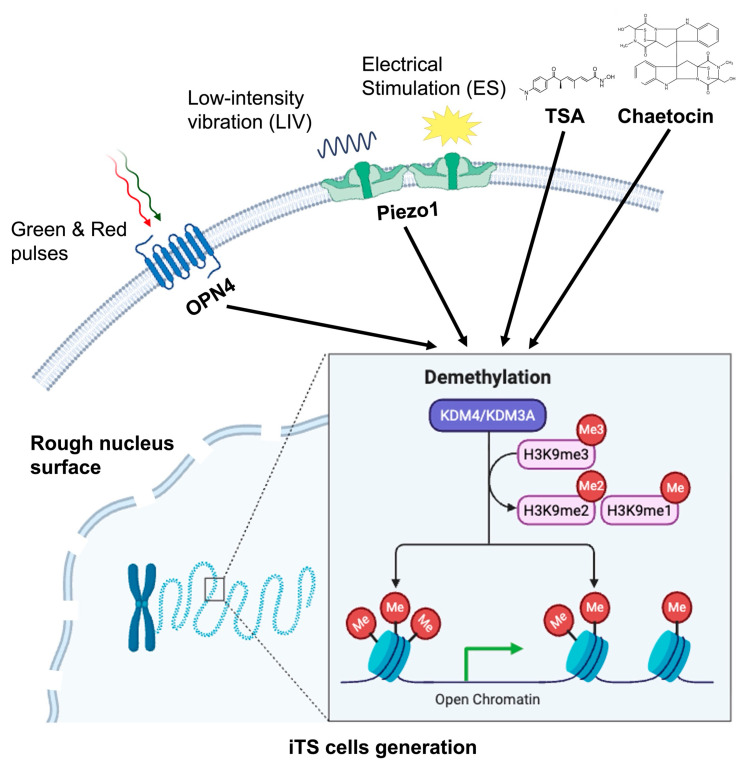
Mechanism underlying the generation of iTS cells in response to biophysical stimuli via chromatin decondensation, including the regulation of H3K9me3 and KDM4.

Immunofluorescence staining confirmed the upregulation of these enzymes (Figure 9A,B). Notably, all six independent iTS cell induction protocols, including two histone-modifying agents (chaetocin and TSA), LIV, ES, and pulsed light exposure (green and red), consistently reduced H3K9me3 levels. This reduction was accompanied by an increase in KDM4, a demethylase with specificity for H3K9me3 (Figure 9C,D). To conceptualize this epigenetic remodeling, we propose a model in which biophysical stimuli promote nucleosome clustering and chromatin reorganization, thereby facilitating the emergence of iTS cells (Figure 10).

## 4. Discussion

This study demonstrated that osteosarcoma cells, including U2OS and MG63 lines, can be reprogrammed into iTS cells through three distinct biophysical stimuli: mechanical vibrations, alternating electrical fields, and visible light pulses. Conditioned media (CM) derived from these iTS cells exhibited tumor-suppressive properties, effectively inhibiting tumor cell progression. Rather than reiterating individual morphological parameters, these findings collectively indicate that biophysical stimulation induces a coordinated nuclear remodeling program. This program is characterized by transient nuclear enlargement, increased nuclear envelope (NE) roughness, and chromatin decondensation. Previous studies have shown that increased nuclear volume is often associated with a more open chromatin configuration that facilitates RNA polymerase II engagement [23], while altered NE topology may influence nuclear pore permeability and transcription factor trafficking [24]. Consistent with these reports, our data suggest that iTS induction is accompanied by a shift toward a more transcriptionally permissive nuclear state. Importantly, pharmacological chromatin relaxation using TSA was sufficient to induce tumor-suppressive CM in the absence of biophysical stimulation, supporting a functional link between chromatin decondensation and iTS-associated phenotypes. However, although nucleosome scattering reproducibly accompanies iTS cell generation, our data do not support a model in which chromatin decondensation acts as a single autonomous driver. Instead, nucleosome scattering is best interpreted as a permissive epigenetic state that facilitates cellular reprogramming.

ITS cells represent a novel cell-based technology designed to inhibit tumor progression [22,25]. Their tumor-suppressive function is mediated primarily through paracrine-like signaling, whereby secreted factors remodel the tumor-supportive microenvironment into a tumor-suppressive one [26]. This concept is consistent with evolutionary models in which rapidly proliferating cells suppress neighboring cell growth to maintain a competitive advantage [27]. Earlier approaches to iTS generation relied on genetic overexpression of oncogenes such as c-Myc, K-Ras, Akt4, β-catenin, Snail, and Oct4 [28], or chemical activation of proliferative signaling pathways including Wnt, PKA, and PI3K [29]. In contrast, the present study demonstrates that non-genetic, biophysical stimulation is sufficient to induce iTS cells, highlighting a distinct and potentially more controllable strategy for tumor-suppressive reprogramming.

Our results further demonstrated that distinct biophysical stimuli engage specific sensory pathways to mediate cellular reprogramming. LIV and ES primarily signal through the mechanosensitive ion channel Piezo1, whereas optical pulses act via the photoreceptive G protein-coupled receptor OPN4 (melanopsin) [30,31]. While our data establish Piezo1 and OPN4 as necessary mediators for their respective stimuli, it is likely that additional mechanosensors and photosensors contribute to the full spectrum of responses. Candidates include TRPV4, integrin-associated complexes, cytoskeletal–nuclear coupling elements, cryptochromes, and other opsins. Thus, iTS cell generation is unlikely to arise from a single linear signaling cascade. Instead, it reflects the integration of multiple sensory inputs converging on chromatin remodeling and tumor-suppressive phenotypes. The precise hierarchy and interaction among these pathways remain to be fully elucidated.

The functional role of nucleosome scattering is also likely to depend on cellular context, including cancer type and developmental state. Excessive chromatin decondensation proved cytotoxic in our system, underscoring the importance of controlled epigenetic modulation. Although this study focused on H3K9me3 as a marker of repressive chromatin, osteosarcoma epigenetics is shaped by a broader network of histone-modifying enzymes, including methyltransferases, deacetylases, and demethylases [32]. For example, H3K27me3, catalyzed by polycomb-associated methyltransferases, is also linked to transcriptional repression [33], while HDACs are frequently upregulated in osteosarcoma and promote chromatin compaction [34]. Demethylases such as KDM6B exhibit context-dependent roles, and loss of SETD2-mediated H3K36me3 has been associated with genomic instability. In this study, TSA was used as a pan-HDAC inhibitor to induce nucleosome scattering and iTS generation, but further work will be required to identify the dominant histone-modifying enzymes governing H3K9me3 dynamics in this context.

A key practical consideration in iTS cell generation is the careful calibration of stimulation parameters and chemical conditions. Insufficient stimulus strength fails to elicit measurable responses, whereas excessive exposure leads to cellular damage. The interplay between stimulus frequency and intensity is particularly critical. While high-intensity or high-frequency stimulation can effectively ablate cancer cells, such conditions are not conducive to iTS induction. Similarly, TSA and chaetocin displayed concentration-dependent, biphasic effects, with moderate doses favoring reprogramming and higher doses inducing cytotoxicity. This behavior mirrors hormetic responses commonly observed in cellular reactions to both chemical and physical stimuli.

We show that biophysical stimulation—via LIV, ES, or optical pulses—remodels the nucleus by enlarging nuclear size, increasing NE roughness, and scattering nucleosomes. These effects are mediated through Piezo1/OPN4 signaling. This aligns with our prior work [30], where Piezo1 activation combined with vibration reduced NE wrinkling and promoted YAP nuclear entry in osteocytes. The mechanism involved coordinated actin–NE dynamics and cytokine signaling. Our findings also echo a decade of NE-focused mechanotransduction studies:

- Nuclear pore dilation accelerates YAP/TAZ import, linking nuclear mechanics to transcription [24].
- NE wrinkling predicts YAP/TAZ localization and mechanosensitive responses in progenitors [35].
- Cytoskeletal forces are transmitted through the LINC complex and lamins, forming a structural bridge from actomyosin tension to chromatin regulation [36].
- Lamin A/C acts as a “mechanostat,” scaling with matrix stiffness to adjust nuclear stiffness and lineage fate [37].
- A- and B-type lamins differentially couple to LINC complexes and cortical filaments, modulating force transmission and nuclear mechanics [38].
- The perinuclear actin cap serves as a high-tension conduit for YAP-driven nuclear mechanotransduction under flow and mechanical load [39].

Together, these studies and our data support a model where cytoskeletal forces reshape NE architecture and chromatin organization. This dynamic remodeling regulates nuclear transport, epigenetic plasticity, and tumor-suppressive programs.

This study has several limitations. First, we focused exclusively on two osteosarcoma cell lines. However, previous research has shown that various tumor and non-tumor cell types, including breast cancer cells, MSCs, and T cells, can also be reprogrammed into iTS cells by LIV and ES. It remains to be determined whether the observed alterations in nucleosome scattering are consistent across a broader range of cell types. Second, our findings are limited to in vitro cell culture models. To evaluate the translational potential of these biophysical stimuli, preclinical studies using animal models are essential. Finally, different biophysical stimuli may induce distinct profiles of tumor-suppressive proteins in the secretome. Future studies should aim to identify which proteins are commonly produced across stimuli and which are unique to specific modalities.

In conclusion, this study integrates morphological quantification of the nuclear envelope, super-resolution analysis of nucleosome clustering, and functional assays in osteosarcoma cells. Together, these approaches reveal a moderate level of chromatin decondensation as a unifying hallmark of iTS reprogramming. This reprogramming can be induced either through chemical inhibition of chromatin condensation or via three distinct biophysical modalities. Notably, biophysical conversion offers the advantage of transience, enabling precise spatial and temporal control through noninvasive controllable devices. This controllability opens new avenues for targeted and dynamic strategies in cancer therapy.

## Data Availability

The datasets generated and analyzed during the current study are available from the corresponding author upon reasonable request. Custom code used for nuclear envelope roughness quantification, L(r)–r analysis, PCA, and STORM point-cloud processing is available from the corresponding author upon reasonable request.

## Supplementary Materials

The following supporting information can be downloaded at: https://www.mdpi.com/article/10.3390/cells15020113/s1.
