# Correction: Xu et al. Nucleosome Clustering as a Biomarker and Mechanistic Switch for Reprogramming Cells. *Cells* 2026, *15*, 113

**DOI:** 10.3390/cells15100894

**Published:** 2026-05-14

**Authors:** Zhaoyuan Xu, Yinzhi Xu, Lidan You, Hiroki Yokota

**Affiliations:** 1 Weldon School of Biomedical Engineering, Purdue University Indianapolis, Indianapolis, IN 46202, USA; xu1990@purdue.edu (Z.X.); xu2019@purdue.edu (Y.X.); 2 Department of Mechanical and Materials Engineering, Queen’s University, Kingston, ON K7L 3N6, Canada; you.lidan@queensu.ca; 3 Indiana University Melvin and Bren Simon Comprehensive Cancer Center, Indianapolis, IN 46202, USA; 4 Department of Anatomy, Cell Biology and Physiology, Indiana University School of Medicine, Indianapolis, IN 46202, USA; 5 Indiana Center for Musculoskeletal Health, Indiana University School of Medicine, Indianapolis, IN 46202, USA

In the original publication [1], the affiliations for Zhaoyuan Xu and Yinzhi Xu should have been indicated as Purdue University only. Baiyan Li and Jing Liu should not be included as authors, and they were removed from the author list. The corrected Author Contributions statement appears here.

**Author Contributions:** Conceptualization, L.Y. and H.Y.; formal analysis, Z.X. and Y.X.; funding acquisition, H.Y.; investigation, Z.X. and Y.X.; project administration, H.Y.; resources, H.Y.; supervision, H.Y.; writing—original draft, Z.X., L.Y. and H.Y.; writing—review and editing, Z.X., L.Y. and H.Y. All authors have read and agreed to the published version of the manuscript.

The authors state that the scientific conclusions are unaffected. This correction was approved by the Academic Editor. The original publication has also been updated.

## References

[1] Xu Z., Xu Y., You L., Yokota H. (2026). Nucleosome Clustering as a Biomarker and Mechanistic Switch for Reprogramming Cells. Cells.

