# Supplementary material for: Nucleosome Clustering as a Biomarker and Mechanistic Switch for Reprogramming Cells
Source: Cells. 2026 Jan 8;15(2):113. doi: 10.3390/cells15020113 (PMC12839345; doi:10.3390/cells15020113)
Supplement: Supplementary file 1 [file cells-15-00113-s001.zip › cells-4057127-supplementary.pdf]

Supplementary Information

Suppl. Table1. STORM Buffer (pH 8.0)

STORM Buffer (1 mL)	890 µL of buffer B<br>10 µL of Cysteamine stock (77 mg/ml in PBS)<br>10 µL of GLOX stock, consisting of 5.6 mg Glucose Oxidase, 80 µl buffer A, and 20 µL Catalase stock (17 mg/ml in PBS)
Cysteamine	30070-10G (Sigma Aldrich)
Glucose Oxidase	G2133-10KU (Sigma Aldrich)
Catalase	C1345-1G (Sigma Aldrich)
Buffer A	10 mM Tris and 50 mM NaCl in 10 ml PBS
Buffer B	50 mM Tris, 10 mM NaCl, and 1g Glucose in 10 ml PBS

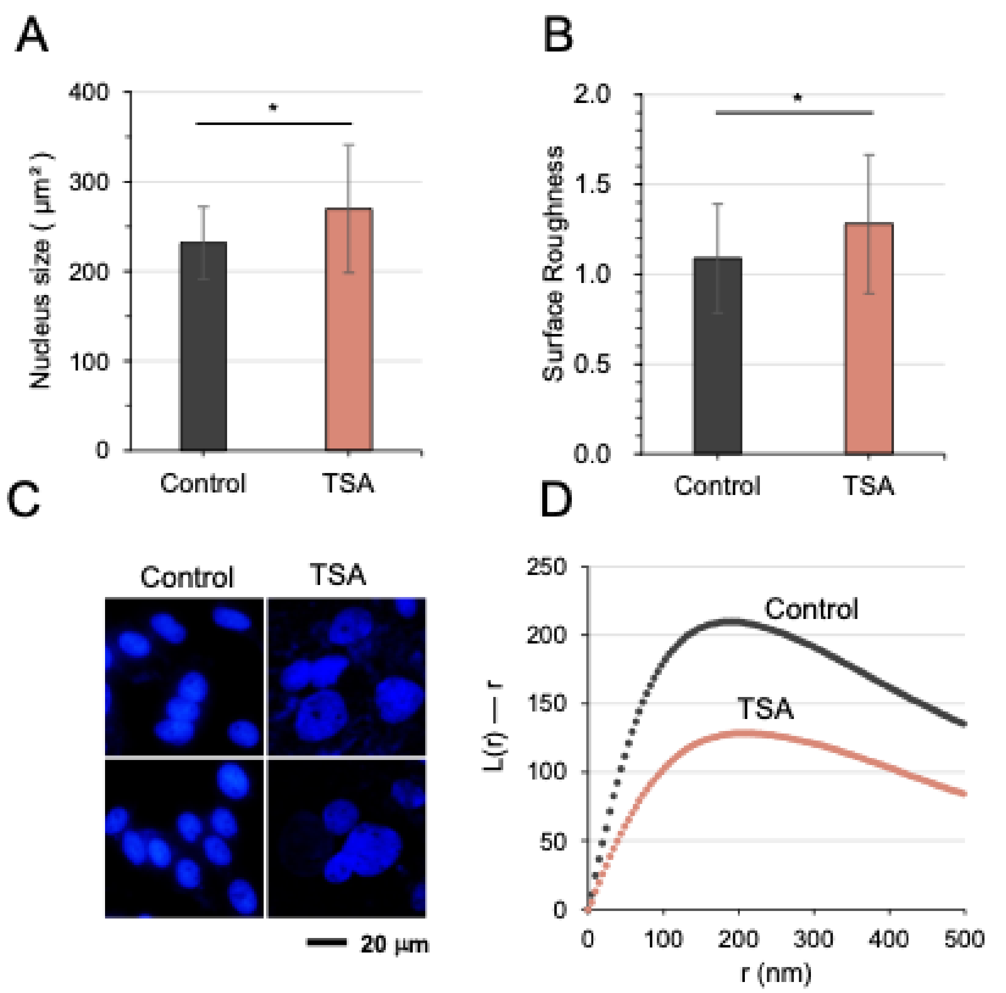

Suppl. Figure 1. Trichostatin A (TSA) induces chromatin decondensation in U2OS cells. TSA, a histone deacetylase (HDAC) inhibitor, promotes a more relaxed chromatin architecture. (A) TSA treatment led to a significant increase in nuclear size in U2OS cells. (B) An increase in nuclear envelope roughness following TSA exposure. (C) TSA treatment resulted in an increased cross-sectional area of DAPI-stained nuclei. (D) A decrease in the “L(r) – r” value, indicative of reduced nucleosome clustering, was observed in response to TSA. The single asterisks(*) indicate $p < 0.05$.

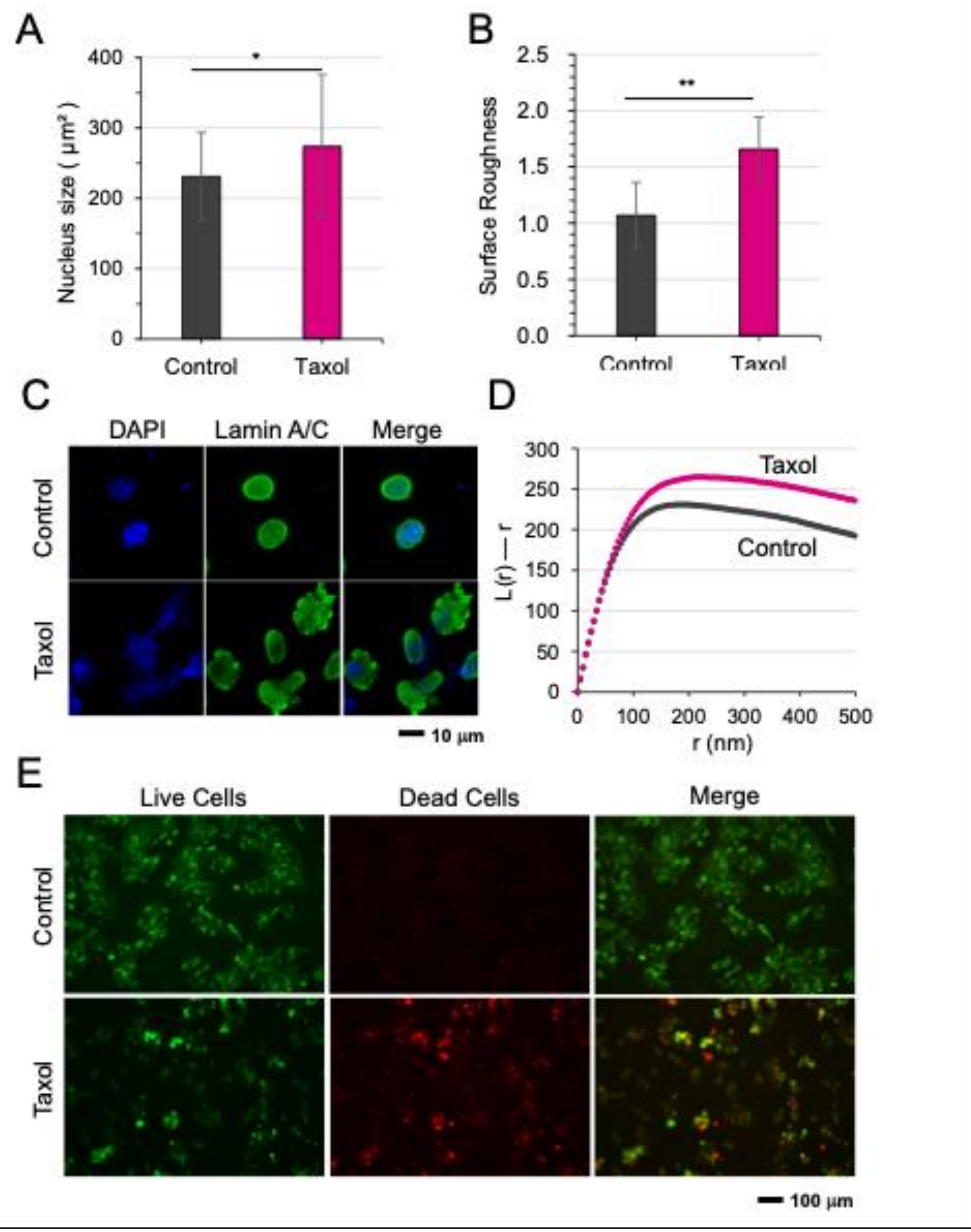

Suppl. Figure 2. Taxol induces cell death and chromatin clustering in U2OS cells. (A) Taxol treatment enlarged the nuclear size. (B) Taxol increased the roughness of the nuclear envelope. (C) Lamin A/C-stained fluorescent images showing nuclear envelope deformation. (D) Taxol enhanced nucleosome clustering. (E) Taxol significantly increased cell death in U2OS cells. Green: live cells; Red: dead cells. The single (*) and double (**) asterisks indicate $p < 0.05$ and 0.01, respectively.

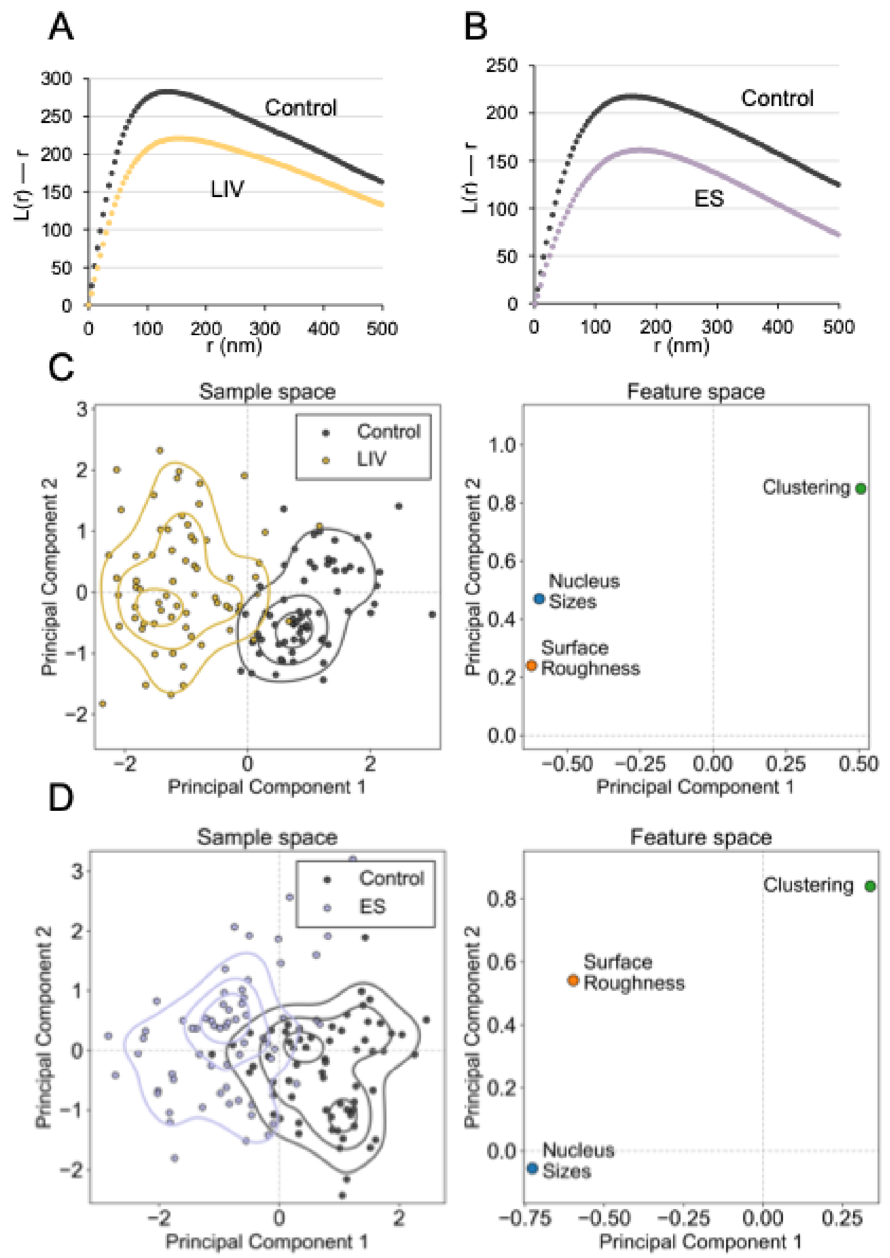

Suppl. Figure 3. Nucleosome clustering and PCA analyses following mechanical vibration and electrical stimulation. (A) Analysis of nucleosome clustering using the L(r) – r function shows a significant decrease in chromatin compaction in U2OS cells following low-intensity vibration (LIV) compared with control cells. (B) Electrical stimulation (ES) similarly reduces nucleosome clustering, as indicated by a decreased L(r) – r value relative to controls. (C) Principal component analysis (PCA) of single-cell measurements following LIV, based on nuclear size, nuclear envelope surface roughness, and nucleosome clustering, shown in sample space (left) and feature space (right). (D) PCA following ES, illustrating the relationships among the same nuclear features in sample and feature space. Together, these analyses demonstrate a negative association between nucleosome clustering and nuclear envelope roughness in response to distinct biophysical stimuli.

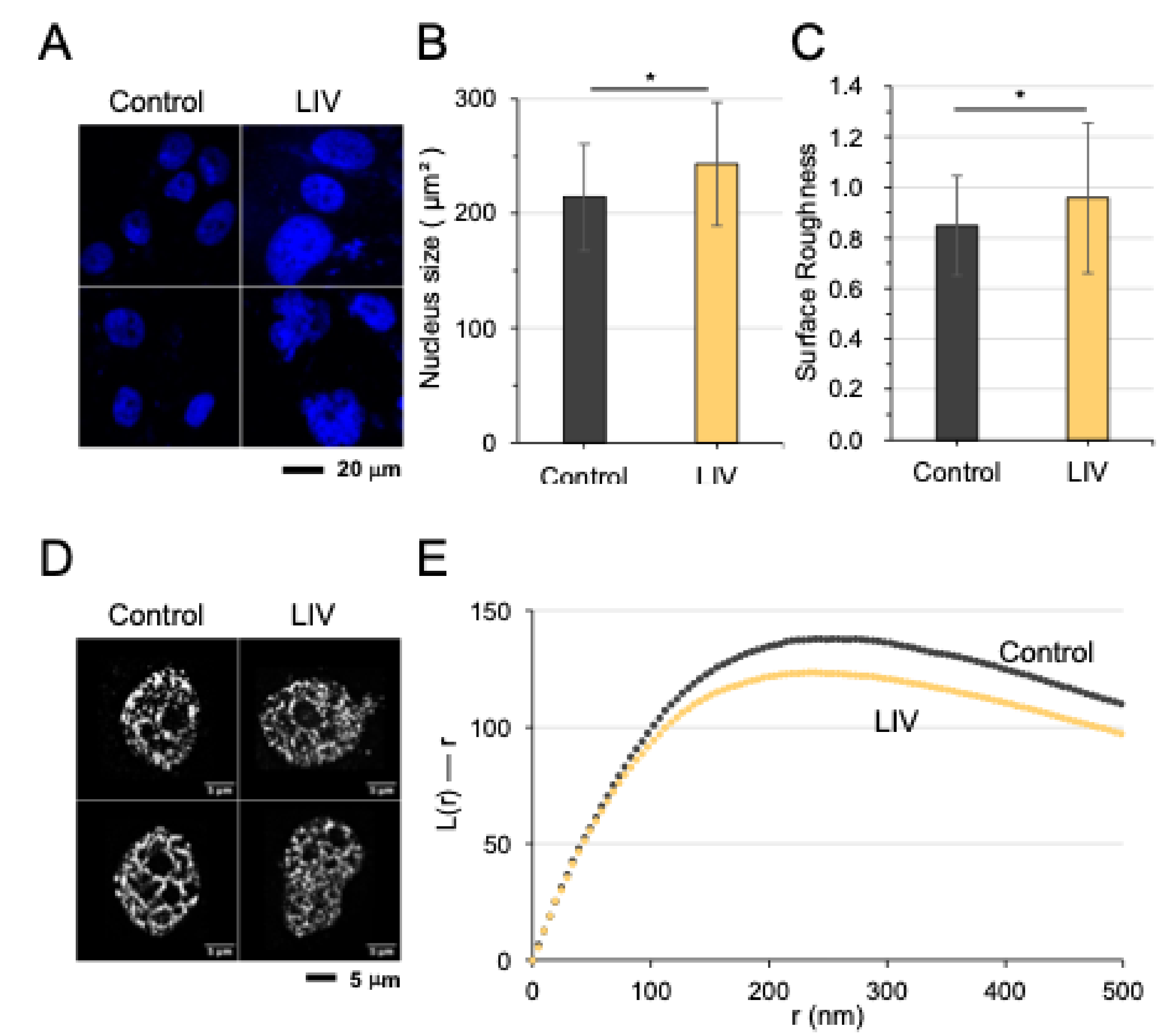

Suppl. Figure 4. Mechanical vibration (LIV – low-intensity vibration) promoted chromatin dispersion and nuclear remodeling in MG63 cells. (A) Representative DAPI-stained images of nuclei before and after LIV, illustrating chromatin dispersion by LIV. (B, C) Quantitative analysis, showing an increase in nuclear size and nuclear envelope surface roughness, following LIV. (D) High-resolution STORM images of nuclei before and after LIV, revealing changes in chromatin organization. (E) Decrease in the "L(r) – r" value after LIV, indicating reduced nucleosome clustering. The single (*) asterisks indicate $p < 0.05$.

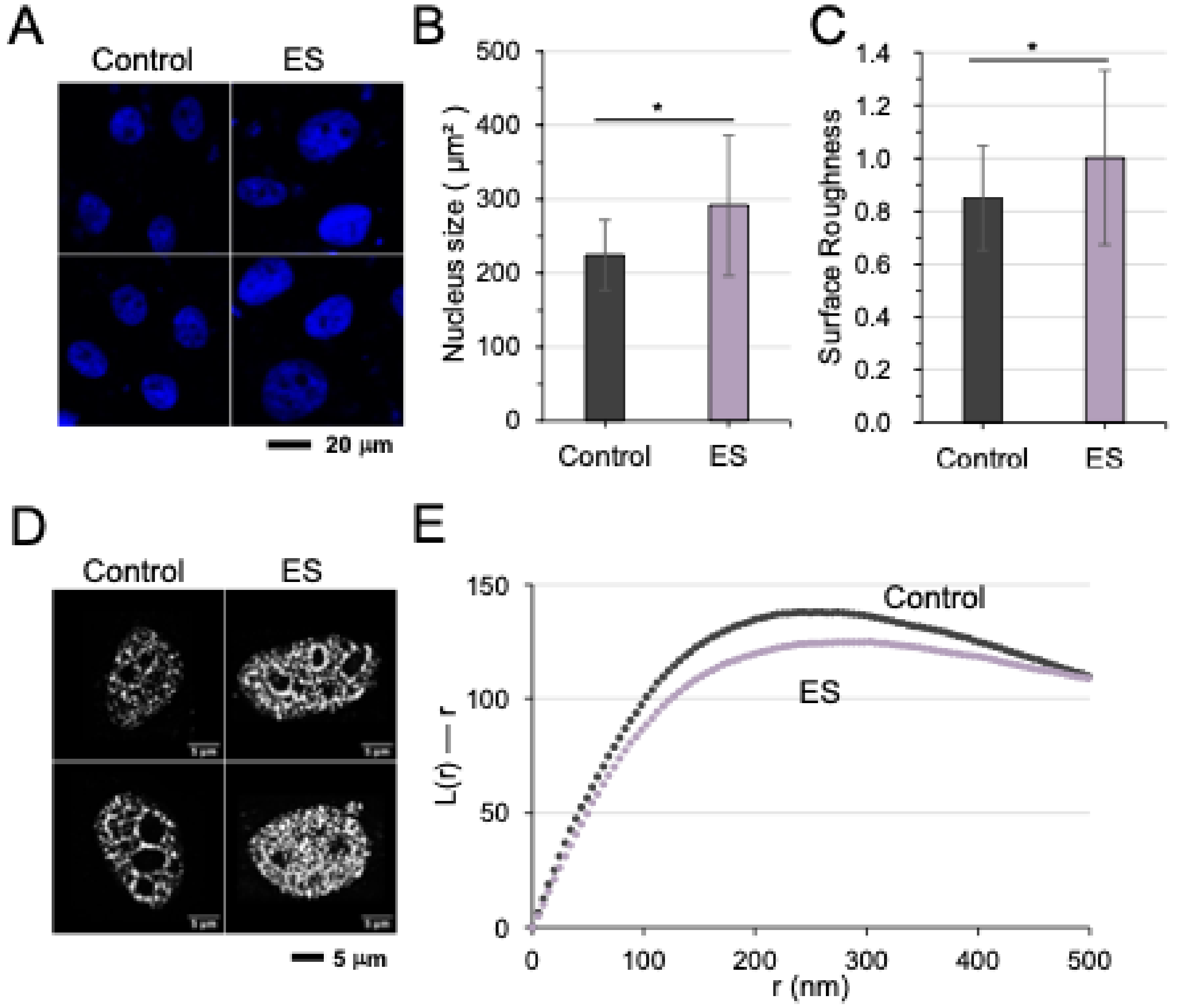

Suppl. Figure 5. Electrical stimulation (ES) promoted chromatin dispersion and nuclear remodeling in MG63 cells. (A) Representative DAPI-stained images of nuclei before and after ES, illustrating chromatin dispersion. (B, C) Quantitative analysis, showing an increase in nuclear size and nuclear envelope surface roughness, following ES. (D) High-resolution STORM images of nuclei before and after ES, revealing changes in chromatin organization. (E) Decrease in the "L(r) – r" value after ES, indicating reduced nucleosome clustering. The single (*) asterisks indicate $p < 0.05$.

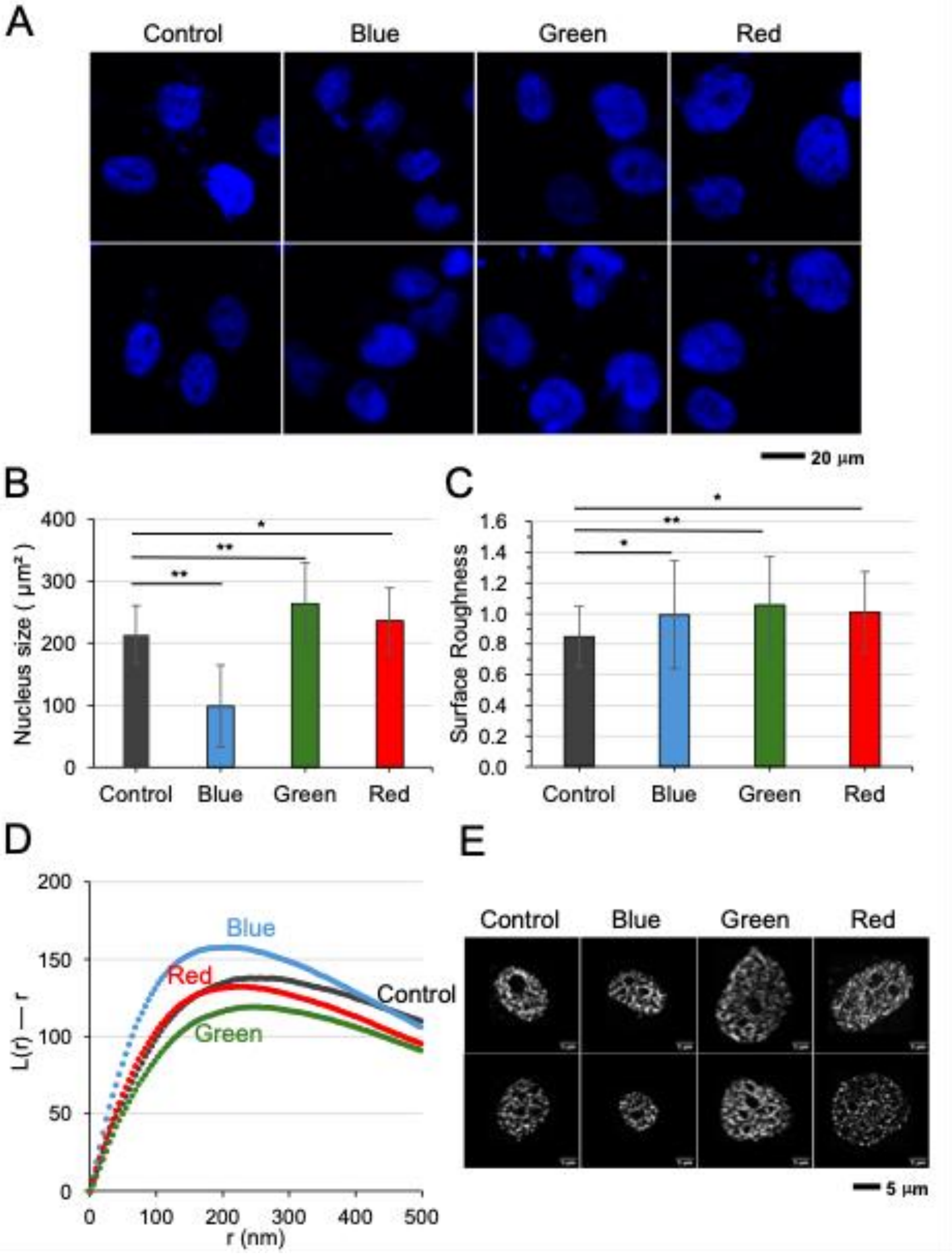

Suppl. Figure 6. Chromatin clustering is modulated by optical pulses in a wavelength-dependent manner in MG63 cells. (A, B) DAPI-stained images of nuclei and quantification of nuclear size changes, following exposure to red, green, or blue light. Red and green light increased nuclear size, whereas blue light caused a reduction. (C) All three wavelengths increased the surface roughness of the nuclear envelope. (D) Red and green light pulses reduced nucleosome clustering, while blue light enhanced clustering. (E) High-resolution STORM images of nuclei before and after irradiation with red, green, or blue pulses, illustrating wavelength-specific effects on chromatin organization. The single (*) and double (**) asterisks indicate $p < 0.05$ and 0.01, respectively.

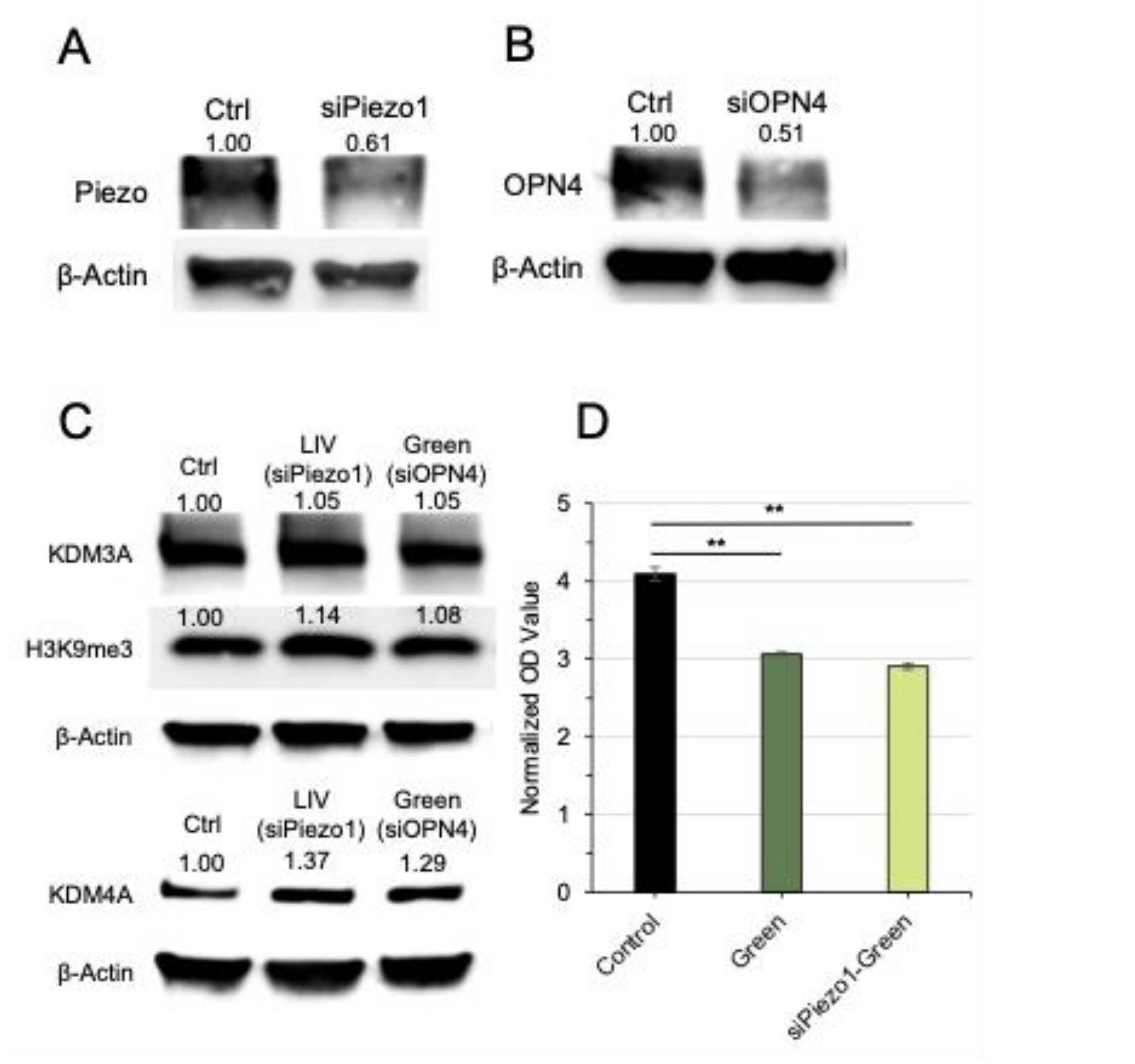

Suppl. Figure 7. Role of Piezo1 and OPN4 in response to LIV/ES and green/red pulses, respectively. (A, B) Silencing of Piezo1 and OPN4, respectively, by RNA interference. (C) Silencing of Piezo1 or OPN4 abolished LIV and green pulse-induced changes in KDM3A, KDM4, and H3K9me3. (D) Piezo1 silencing does not block the tumor-suppressive effect of the conditional medium from green pulse-treated iTS cells. The double (**) asterisks indicate $p < 0.01$.
